# Scalable spokes pTx pulses for 2D turbo‐spin‐echo imaging at 7 T

**DOI:** 10.1002/mrm.70068

**Published:** 2025-09-29

**Authors:** Minghao Zhang, Christopher T. Rodgers

**Affiliations:** ^1^ Wolfson Brain Imaging Centre, University of Cambridge Cambridge United Kingdom

**Keywords:** fast spin echo, parallel transmit, scalable pulses, spokes, turbo spin echo

## Abstract

**Purpose:**

Turbo spin echo (TSE) is important clinically. Unfortunately, 7 T TSE suffers from B_1_^+^‐induced signal dropouts. Magnitude‐based parallel transmit (pTx) pulse design algorithms cannot enforce phase patterns complying with the Carr‐Purcell‐Meiboom‐Gill conditions (90° phase shift between excitation and refocusing). We introduce scalable spokes pTx pulses for 7 T TSE imaging.

**Theory:**

We define *scalable spokes pulses* as having time‐symmetric RF waveforms, antisymmetric in‐plane gradients, and rephased subpulse slice‐selection gradients. They produce flip angles that are approximately proportional to the applied voltage with voltage‐independent phase patterns.

**Methods:**

Scalable spokes pulses were designed for a phantom. Scaling behavior was characterized via Bloch simulations. Performance in terms of TSE echo homogeneity was assessed by extended phase graph simulations using in vivo field maps.

Performance was validated for TSE acquisitions in a phantom and in vivo. Hippocampal TSE imaging was performed for four subjects comparing circularly polarized (CP), RF shimming, and scalable spokes pulses.

**Results:**

Scalable spokes pTx pulses show similar scaling behavior to previously proposed 3D k_T_‐point pulses. Scalable three‐spoke pulses decrease flip‐angle RMS error across subjects compared to CP mode pulses (11% vs. 23% for 120° pulses). TSE images with these pulses recover signals in cerebellum and temporal lobes.

**Conclusion:**

Scalable spokes pTx pulses produce flip angles that vary approximately linearly with peak voltage while maintaining consistent spatial patterns of phase. Together with their spatial flip‐angle homogeneity, these pulses enable high‐fidelity 2D slice‐by‐slice TSE imaging at 7 T, albeit with reduced slice coverage with our choice of homogeneity target under the current vendor‐provided specific absorption rate constraints on 7 T MRI scanners.

## 1 INTRODUCTION

Turbo spin echo (TSE, also known as RARE or FSE)^1^ is the most commonly used sequence for clinical T_2_‐weighted structural imaging. It produces images with clinically relevant contrast and high SNR from a short scan. TSE of the brain is important for clinical assessment of patients with multiple sclerosis or hippocampal atrophy.^2^, ^3^, ^4^, ^5^

**FIGURE 1:**
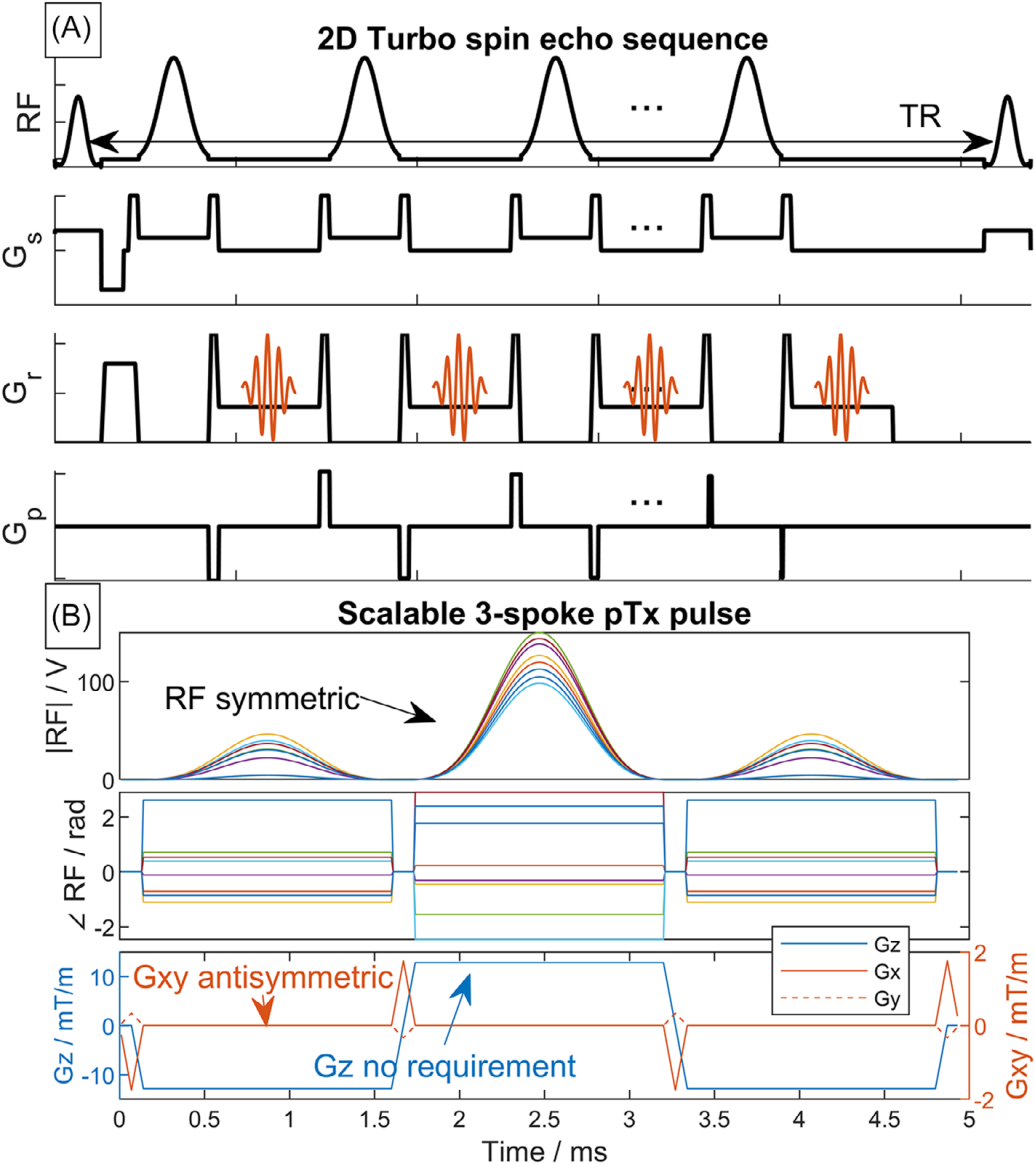
(A) Schematic pulse sequence diagram for a 2D TSE sequence. (B) Example of waveforms for a scalable three‐spoke pTx pulse. The RF waveforms are symmetric in time about the midpoint of the pulse, and G_xy_ is antisymmetric. The slice‐selection gradient G_z_ for three spokes can be symmetric for more degrees of freedom to tackle RF field inhomogeneity compared to two spokes, at the expense of possibly reduced scalability. pTx, parallel transmit; TSE, turbo spin echo.

Ultrahigh field 7 T MRI improves spatial resolution and image SNR compared to typical hospital 1.5 T or 3 T MRIs.^6^, ^7^ These benefits accrue also for TSE imaging, which has proven to be important for identifying small structural abnormalities such as in patients with epilepsy.^8^ However, UHF MRI often suffers from regions of signal dropout or contrast loss due to B_1_^+^ field inhomogeneities.^9^ TSE is particularly affected due to its repeated refocusing pulses (Figure 1). High flip angle pulses are more severely affected by the inhomogeneous B_1_^+^ field, and they generate different amounts of spin echo and stimulated echo pathways across the imaging volume, resulting in different contrasts. As a result, 7 T TSE images are often not of diagnostic quality in the inferior brain (temporal lobes, cerebellum, brainstem).^10^

The effect of imperfect refocusing in TSE is minimized providing that the Carr‐Purcell‐Meiboom‐Gill (CPMG) condition^11^, ^12^ is satisfied: There must be a 90° phase shift at each voxel between the phase of the excitation pulse and the refocusing pulses. When the CPMG condition is met, the resulting train of echoes has consistent phases, and the effects of imperfect refocusing pulse flip angles are reduced.

TSE sequences can be implemented with 2D or 3D readouts.^13^, ^14^ 3D TSE scans usually collect a larger number of echoes than 2D scans after each excitation to increase the acquisition efficiency. Whereas some neuroimaging applications favor 3D TSE,^15^ in a recent pilot study for presurgical assessment of patients with severe epilepsy, the neurologists and neuroradiologists at our center subjectively preferred slice‐by‐slice 2D TSE for hippocampal assessments because of its better sharpness.^8^

The additional degrees of freedom provided by parallel transmit (pTx) hardware can be used to improve image homogeneity, which especially improves inferior brain for whole‐brain imaging protocols.^16^, ^17^, ^18^ TSE sequences must respect the CPMG condition, and yet typical pTx pulse design approaches are based on magnitude least squares^19^ optimization. If this is applied separately to the excitation and refocusing pulses, they are highly unlikely to satisfy the CPMG criteria (and then only for certain voxels), leading to reduced echo intensities and producing severe image artifacts. One solution is to treat the whole refocusing train as a single optimization problem and apply direct signal control (DSC) to directly optimize the echo signals with RF shimming, which is the approach introduced by Malik et al. and applied in the popular DiSCoVER work‐in‐progress sequence package (Siemens Healthcare, Erlangen, Germany).^20^, ^21^ DiSCoVER optimizes the RF shims of the excitation pulse and all refocusing pulses in the echo train simultaneously, designing with one slab covering the volume of interest. To exploit the capability of dynamic pTx, Yetisir et al. recently proposed a form of 2D TSE in which they enforced a phase‐matching between the excitation and refocusing pulses during pulse design.^22^ This is an appealing approach because the two‐spoke pulses achieved better flip‐angle uniformity than RF shimming and the authors implemented rapid optimization code on GPU. However, to our knowledge, spokes position optimization was not included, and so far it has only been demonstrated in phantoms.

For 3D TSE with k_T_‐points nonselective pulses, Eggenschwiler et al.^23^ introduced a method of *scalable pulses* whereby a single pulse design can be applied with different peak voltage for use as both an excitation and a refocusing pulse. The scalable behavior arises due to certain constraints on the RF and gradient waveform temporal symmetry (described below and explained by Gras et al.^24^), which means that the pulse maintains practically the same phase profile across the imaged volume to satisfy the CPMG criteria. This concept has been extended to more sophisticated waveforms with both universal^25^, ^26^ and subject‐specific optimizations.^27^

In this paper, we introduce slice‐selective 2D scalable spokes pTx pulses for excitation and refocusing in TSE imaging. We analyze the scaling properties of these pulses in simulations, validate their feasibility with phantom scans, and assess their impact on 2D TSE imaging in vivo.

## 2 THEORY

Neglecting off‐resonance and relaxation, an amplitude‐modulated pulse (e.g., rect or sinc pulse) obeys:

(1) $$\alpha \left(r\right)\propto \tilde{V},$$

where α(r) is the complex flip angle (magnitude and phase) at point r, and $\tilde{V}$ is a complex phasor denoting the overall voltage scaling and phase shift of the pulse envelope. In other words, the flip angle |α| is directly proportional to the applied voltage $\left|\tilde{V}\right|$, and the phase of the resulting magnetization stays constant if $∠\tilde{V}$ does not change. Composite pulses do not in general obey Equation (1), but a special set of scalable pulses do so approximately. A scalable pulse can be applied for both excitation (at $\tilde{V}$) and refocusing (at approximately 2× $\tilde{V}$) while maintaining a consistent phase difference between excitation and refocusing for all points across the volume of interest.

Gras et al.^24^ showed that a sufficient condition for a pulse to be scalable (to first order of the Magnus expansion of the spin Hamiltonian in average Hamiltonian theory),^28^ is that its RF waveform is symmetric in time about the middle of the pulse, the gradient waveform is antisymmetric about that timepoint, and ΔB_0_ offset effects are negligible.^23^ Furthermore, when accounting for ΔB_0_ offset, shorter pulses scale more faithfully and additional pulse design algorithm refinements can further promote scalability.^24^

We present a similar reasoning that is applicable to slice‐selective spokes pulses.^29^, ^30^

To apply average Hamiltonian theory (see Brinkmann^28^ for a review of the theory), we consider a spin one‐half particle at position r. In the rotating frame, its Hamiltonian is:

(2) H(t)=12(Δω+γG(t)·r)σz+12Reω1(t)σx+Imω1(t)σy,

where Δω is the off‐resonance frequency, γ is the gyromagnetic ratio, G(t) is the gradient waveform vector, $\sigma_{x,y,z}$ are the Pauli matrices (representing the *x*, *y*, *z* spin rotation operators), and $\omega_{1}\left(t\right)=\gamma B_{1}\left(t\right)$ is the complex RF waveform. We partition the Hamiltonian $H\left(t\right)=H_{0}\left(t\right)+H_{1}\left(t\right)$ to move into an interaction frame and leave the *z*‐rotation terms in the free Hamiltonian $H_{0}$. The interaction Hamiltonian is therefore

(3) HI(t)=12Ree−i(Δωt+(k(t)−k(0))·r)ω1(t)σx+Ime−i(Δωt+(k(t)−k(0))·r)ω1(t)σy,

where $k\left(t\right)=\gamma \int_{T}^{t}G\left(t^{\prime }\right)\;dt^{\prime }$ is the transmit k‐space location. First, consider one subpulse, that is, a single frequency‐selective pulse and its associated slice‐select gradient as shown in Figure S1A. The leading order approximation in its Magnus expansion is

(4) HI‾(0)=12Re{θ‾(z)}σx+Im{θ‾(z)}σy,

where $\bar{\theta }\left(z\right)=\int_{0}^{T}e^{-i\left(\Delta \omega +\gamma G_{z}z\right)t}\omega_{1}\left(t\right)\mathrm{d}t$, $G_{z}$ is the slice‐selection gradient amplitude, T is the pulse duration, and is the slice off‐center position. $\bar{\theta }\left(z\right)$ is proportional to the overall $\omega_{1}$ scaling of the pulse and hence is the rotation $\left|\bar{\theta }\left(z\right)\right|$ about an axis $\arg\left(\bar{\theta }\left(z\right)\right)$ in the transverse *xy* plane. Note that this is not equivalent to the linear perturbation theory on the magnetization; instead the Hamiltonian, and hence the angle, is applied in the argument of an exponential, explaining why the small tip angle (STA) approximation stays valid even at relatively high flip angles.^31^ First‐order terms A^(1)^ contribute an additional *z* component to the equivalent rotation that scales as the overall $\omega_{1}^{2}$.^32^ Second‐order and higher terms are very small for typical spokes subpulses. In other words, to the leading order approximation, the effect of one slice‐selective pulse in the interaction frame is equivalent to an instantaneous *z*‐dependent rotation about a transversal axis, whose angle is given by the STA formula. This can be written as a propagator, or rotation matrix in SU (2),

(5) $$\begin{array}{c} U_{\mathrm{sub}}^{\left(0\right)}\left(z\right)=\exp\left(-\frac{i}{2}\left(\left|\bar{\theta }\left(z\right)\right|\sigma_{\arg\left(\bar{\theta }\left(z\right)\right)}\right)\right). \end{array}$$

We can construct subpulses as shown in Figure S1C by adding pre‐winder and re‐winder gradients with half the gradient moment of the slice‐selection gradient. These make each subpulse self‐refocused. It allow us to use the result from Gras et al.,^24^ that when transforming back to the original rotating frame, the propagators can be decomposed into the instantaneous *z*‐dependent transverse rotation sandwiched between a free precession for half of the pulse duration (Figure S1D), $U_{\text{unit}}^{\left(0\right)}=R_{\mathrm{fp}}U_{\mathrm{sub}}^{\left(0\right)}\left(z\right)R_{\mathrm{fp}}$ with $R_{\mathrm{fp}}=e^{-i\Delta \omega T\sigma_{z}/4}$.

When the spokes pulse is played in the bipolar form (i.e., neighboring subpulses use opposite $G_{z}$ polarities), the moments between spokes can be timed to overlap and cancel. Note that the RF waveform is conjugated for subpulses with negative $G_{z}$ gradients, so they produce the same flip‐angle profile $\bar{\theta }\left(z\right)$ if $\Delta B_{0}$ effects are small and the subpulses share the same symmetric slice profile. Under this formalism, we can consider the overall spokes pulse propagators for a position *z* as a list of instantaneous transverse rotations ${\bar{\theta }}_{n}\left(z\right)$ whose angle and axis depend on *z*, interleaved with free precessions due to $\Delta B_{0}$ (Figure S1F). The $G_{z}$ gradients are helpfully eliminated from our analysis.

We then apply the symmetry criteria of Gras et al.^24^ to this composite pulse. If the overall RF ${\bar{\theta }}_{n}$ is symmetric in time and the remaining gradients (i.e., the *xy* spokes gradient blips) are antisymmetric in time, the rotation effect of such a composite pulse can be approximated again by the STA rotation angle up to the first order of Magnus expansion; that is,

(6) $$\begin{array}{c} {\bar{\theta }}_{\text{spokes}}\left(z\right)\approx \sum_{n=1}^{n_{\text{spoke}}}e^{-i\left(\Delta \omega t_{n}+\left(k_{\mathrm{xy}}\left(t_{n}\right)-k_{\mathrm{xy}}\left(0\right)\right)\cdot r\right)}{\bar{\theta }}_{n}\left(z\right), \end{array}$$

where $k_{\mathrm{xy}}\left(t\right)=\int_{T}^{t}\gamma G_{\mathrm{xy}}\left(t^{\prime }\right)\mathrm{d}t^{\prime }$ is the in‐plane transmit k‐space trajectory, $t_{n}$ is the time at the center of the *n*th‐spoke. In other words, the leading order approximation of such composite spokes pulses is a single rotation that is proportional to the overall $B_{1}^{+}$ amplitude and has a purely transverse axis of rotation in the interaction frame for every z position, if $\Delta B_{0}$ is small. The slice profile (i.e., *z*‐dependence of ${\bar{\theta }}_{\text{spokes}}$) is approximately the same as that of a single subpulse. Similar to the single pulse case, when moving out of the interaction picture back to the initial rotating frame, this rotation is sandwiched by $\Delta B_{0}$ precessions,^24^ thereby explaining within the limits of the approximation the scaling and desired refocusing properties of such RF pulse.

From this analysis, we conclude that a 2D slice‐selective spokes pulse will be approximately scalable if it has a:

1. symmetric RF waveform around pulse midpoint, that is, symmetric subpulse RF coefficients and the same RF subpulse waveforms for each spoke, and
2. antisymmetric in‐plane gradients, that is, symmetric spokes positions in transmit k‐space, and
3. subpulse RF waveform and corresponding balanced slice‐selection gradients where all subpulses achieve approximately the same slice profile.

This means that the available degrees of freedom (DOFs) in an $n_{\text{spoke}}$‐spoke pulse reduce to match a $\text{ceil}\left(n_{\text{spoke}}/2\right)$‐spoke pulse. For pulses with a bipolar gradient form, an odd number of spokes always implies a symmetric G_*z*_ form, and vice versa for even numbers of spokes. Unlike the hard‐pulse subpulses used in 3D kT‐point pulses,^33^ which last for a few hundred microseconds each, even at high flip angles, slice‐selective subpulses typically need more than 1 to 2 ms to achieve a reasonable slice profile while satisfying peak voltage constraints. Therefore, the possibility to neglect the overall G_*z*_ symmetry derived above is of practical importance. For example, it allows a three‐spoke pulse, which provides extra DOFs from scalable two‐spoke pTx pulses (which only have the same DOFs as RF shimming) but can be fitted in a ˜5 ms overall duration at a possible expense of scalability.

An example form of a scalable three‐spoke pulse is shown in Figure 1B. Note that for refocusing pulses (e.g., in TSE), it is important to include both pre‐ *and* post‐pulse in‐plane gradient blips to ensure that the in‐plane gradient moments are balanced so that echoes are refocused properly in‐plane. The prephasing and rephasing *z*‐gradient can be absorbed into the crushers.

Overall, for simplicity, we performed the analysis for each spoke subpulse separately to explain the *z*‐dependent behavior and maintain a transverse rotation axis with scalable flip angle. This was achieved by moving into an interaction frame suppressing the G_*z*_ contribution in the Hamiltonian, leaving its influence only on the flip angle (described by STA formula). A second analysis on the overall composite shape was performed as if it was nonselective, just as for kT‐points (Figure S1).^24^ A similar analysis could likely be performed only once on the full composite shape.

It should be noted that for even‐numbered spoke pulses, the antisymmetry of the gradient waveforms results in a total absence of longitudinal components in the Magnus expansion of $H_{I}$, and thus no first‐order term when the $\Delta B_{0}$ offset is 0,^24^, ^32^ favoring more scalability compared to an odd‐numbered spoke pulse. For further reading, one paper by Levitt in fact provides an elegant demonstration that any palindromic sequence yields a purely transverse rotation axis,^34^ which is thus the case for symmetric and antisymmetric gradient waveforms for even‐numbered spoke pulses when there is no $\Delta B_{0}$ offset.

## 3 METHODS

### 3.1 Pulse design

Pulse optimizations were performed in MatLab (version 2024a; MathWorks, Natick, MA) using a modified version of the PTx Pulse Design (PPD) package (Siemens Healthineers). Spoke parameters were optimized with Bayesian optimization of gradient trajectory^35^ and an inner loop of a Tikhonov‐regularized magnitude least‐squares RF optimizer^19^ with virtual observation point (VOP)^36^ constraints. The Bayesian optimization was performed with MatLab (version 2024a; MathWorks) bayesopt function, and the constrained optimization with fmincon. The optimization is a variation from the original Bayesian optimization of gradient trajectory implementation and can be summarized as follows:

(7) κ^=argmin|A(κ)b^|−atarget2+λ‖b^‖2,whereb^=argmin|A(κ)b|−atarget2+λ||b||2for fixedκ,

$$\text{subject to}\;b^{*}S_{v}b\leq {\text{LSAR}}_{\max}\forall v\in \mathrm{VOP},\left|b_{i}\right|\leq V_{\max,i}$$

where A is the design matrix (encoding B_1_^+^ sensitivity, B_0_, and subpulse waveform information), b represents the spoke RF coefficients, κ is the spoke position, $a_{\text{target}}$ is the (real) target flip angle,^31^ λ is a Tikhonov regularization factor, $S_{v}$ is the vth patient‐protection VOP matrix, ${\text{LSAR}}_{\max}$ is the local specific absorption rate (SAR) limit (e.g., 20 W/kg for IEC 60601–2‐23 third revision for head), and $V_{\max}$ is the maximum channel voltage.^37^ In our system, the local SAR limit (1 W/channel implemented by the vendor as diagonal VOPs) dominates the global SAR and coil protection limits; thus, the latter were not included.

The symmetry requirements were enforced by explicitly setting the RF coefficients $b_{i}=b_{n_{\text{spoke}}-i+1}$, and the spoke positions $\kappa_{i}=\kappa_{n_{\text{spoke}}-i+1}$, where $i=1,2,\ldots \text{floor}\left(n_{\text{spoke}}/2\right)$.

All pulse designs were performed on CPU on a desktop computer (Intel Core i9‐10900X, 64 GB RAM).

### 3.2 Scalability simulations

Simulations were performed on phantom and in vivo field maps collected from our 7 T Terra MRI (Siemens Healthineers) and 8Tx32Rx head coil (Nova Medical, Wilmington, MA), using the vendor‐provided magnetization‐prepared turbo‐FLASH (satTFL)^38^ B_1_^+^ and dual‐echo gradient echo B_0_‐mapping sequences. They were processed, masked, and exported as MatLab (version 2024a; MathWorks) files in the vendor's PPD framework (Siemens Healthineers). A further linear correction suggested by Tomi‐Tricott et al.^39^ was applied to all satTFL B_1_^+^ maps in this paper.

To test pulse scalability (i.e., compliance with Equation 1), three‐spoke pulses with and without symmetry constraints were designed on a set of phantom field maps for a 120° flip angle. Other design parameters were kept the same. On the resulting pulses, the voltage was then scaled between 0.67× and 1.33× (i.e., for nominal flip angles between 80° and 160°), after which the pulse performance was computed with Bloch simulations including off‐resonance effects.

Two types of error metrics were computed. One is the scaling error, which calculates how the complex flip angle pattern deviates from ideal linear scaling of the actual designed flip angle. This can be broken down into the pixelwise magnitude and phase errors:

(8) $$\begin{array}{c} \epsilon_{\text{scaling},\mathrm{mag}}=\left\|\frac{1}{\alpha_{\text{eval}}}\left(\left|a_{\text{scaled}}\right|-\frac{\alpha_{\text{eval}}}{\alpha_{\text{designed}}}\left|a_{\text{designed}}\right|\right)\right\|, \end{array}$$

(9) $$\begin{array}{c} \epsilon_{\text{scaling},\mathrm{pha}}=\left\|e^{i∠a_{\text{scaled}}}-e^{i∠a_{\text{designed}}}\right\| \end{array}$$

where α denotes the nominal flip angle (a single real‐valued scalar), and a(r) denotes the complex flip angle pattern of the pulses evaluated from Bloch simulations. The phase error measures the distance on a unit circle so that positive and negative errors do not cancel out. The outer norm is taken over all voxels.

The other metric describes complex flip‐angle error against the target flip angle. The phase of the target takes that of the designed pattern. The error is measured as the Euclidean distance of magnetization on the Bloch sphere:

(10) $$\begin{array}{c} \epsilon =\left\|B\left(a_{\text{scaled}}\right)-B\left(\alpha_{\text{target}}e^{i∠a_{\text{designed}}}\right)\right\|, \end{array}$$

where B(.) maps a complex flip angle to the point on Bloch sphere when flipped from equilibrium (Mz = 1).

The rotation axis was also computed from Bloch simulations to evaluate the deviation from a purely transverse rotation after undoing the net B_0_ sandwiching evolutions predicted by the theory.^24^, ^32^

To further investigate whether the choice of design flip angle affects the scaling quality or the range of flip angles over which a single pulse can be scaled robustly, scalable three‐spoke pulses were designed for a range of flip angles (10°, 30°, 60°, 90°, 120°, and 150°). Each was scaled to flip angles between 10° and 180° at 10° intervals, and the scaling errors were calculated.

Bloch simulations were also performed to test whether the symmetric slice‐selection gradients, instead of the overall antisymmetry form derived in the previous paper,^24^ impaired the pulse performance. One midbrain slice of in vivo field map with ΔB_0_ in range (−100.8 Hz, 55.2 Hz) was used. The flip angles (including phase) of the scaled pulses (designed for 120° and scaled between 80° and 160°, same range as above) were simulated in the through‐slice direction at 0.1 mm resolution. The results were compared to two‐spoke and four‐spoke pulses, which obey the antisymmetry requirement, designed with the same algorithm and parameters. Slice profiles were compared quantitatively in terms of the variation of FWHM across all flip angles in all voxels.

### 3.3 Extended phase graph simulations

Spatially resolved extended phase graph (EPG) simulations were performed to predict the echo amplitudes using the 2D scalable spokes pulses in a TSE sequence.^40^, ^41^

A 120° scalable three‐spoke pulse was designed for a set of in vivo ΔB_0_ and per‐channel B_1_^+^ field maps. The pulse was scaled to 90° and 180°, and a 90° phase shift was applied to the refocusing pulse for CPMG compliance. Bloch simulations starting from equilibrium magnetization were used to approximate the spokes pulse as an instantaneous rotation matrix, that is, neglecting T_1_ and T_2_ during the pulse. This instantaneous approximation was used for EPG simulation of a TSE sequence (shown in Figure 1) comprising nine echoes with 12 ms echo spacing, 1000 ms T_1_, and 100 ms T_2_. The EPG simulations of the same sequence were performed for the conventional circularly polarized (CP) pulse for comparison, where the pulses were modeled from the combined B_1_^+^ maps adjusted by the reference voltage, without using a Bloch simulation.

We ran a DSC RF‐shimming optimization^21^ for the same echo train to further compare the performance of our pulse against a popular pTx method. The computation was performed in MatLab (version 2024a; MathWorks) using open‐source code from the original authors. The shim weightings of the excitation and all refocusing pulses were allowed to vary individually. We allowed a maximum of 50 iterations. Total and per‐channel power was constrained to 2.0 times the respective values for the CP sequence to allow a reasonable increase in uniformity.

### 3.4 Phantom validation

Measurements were performed on our 7 T Terra MRI (Siemens Healthineers). The vendor's TSE sequence was modified to allow spokes pTx pulse excitation and refocusing with any user‐defined subpulse waveform through a custom “sequence‐building block” (SBB). This SBB includes trim‐blip correction^42^ for the RF‐gradient timing delay, which can negatively impact the performance of bipolar spokes pulses unless corrected.^43^, ^44^

We calibrated timing delays specifically for the scalable three‐spoke pTx pulse in our TSE protocol using the principle of image nulling.^44^ The TSE sequence parameters were configured to the desired protocol (described below), the spoke blips were set to all zeroes, and the spoke coefficients were set as follows: CP mode for the first spoke, circularly polarized mode with 180° extra phase for the second spoke, zeroes for the third spoke. For an ideal result, all slices should have no signal as the effect of the two pulses should cancel exactly. The measurements were performed with straight slice positioning in the three gradient directions in turn, starting from previously obtained system calibration values. The eddy current phase compensation was adjusted first so that there was minimal signal in the slice at isocenter. Then, the gradient timing delay of that axis was adjusted until there was minimal variation across slices. On our system, the delays in the *x*,*y*,*z* axes were measured to be 4.0, 2.5, 3.0 μs, with −10° eddy current compensation for the three‐spoke pulses specified below.

A scalable three‐spoke 120° pTx pulse was designed as described above for the UK7T study's spherical agar phantom.^45^ The subpulse form used in all TSE scans is a Gauss pulse (as used in vendor's TSE sequence for the “low SAR” option). The subpulse duration is 1.6 ms and the total three‐spoke duration is 5.28 ms, which is consistent with the vendor's low SAR TSE refocusing pulse option. To reduce SAR further, variable‐rate selective excitation (VERSE)^46^ was applied after pulse optimization without changing the duration of subpulses. This has no impact on the symmetry requirements. We did not perform VERSE conversion for CP mode (and RF shimming, below) pulses because the slice‐selection gradient amplitude is already low (˜5 mT/m, compared to ˜9 mT/m for VERSE'd three‐spoke pulse) due to the long pulse‐duration amplitude for the CP mode pulse. VERSE conversion would further reduce its off‐resonance robustness.

TSE images were acquired with the scalable three‐spoke pTx pulse and in CP mode as reference.

The first test used a standard TSE train with 90° excitation, constant 120° refocusing (except for the first refocusing pulse, which was a 150° “stabilizing” pulse^47^), 76 ms TE, 5000 ms TR, 0.9 mm in‐plane resolution, 12.8 ms echo spacing, nine echoes per shot, and 3× GRAPPA acceleration.

In the second test, further images were acquired with a hyperecho train^48^ (with minimum flip angle 120°) to demonstrate the use of scalable pulses in a variable flip angle TSE.

### 3.5 In vivo validation

We compared TSE performance for high‐resolution hippocampal imaging between CP, DSC with RF shimming, and scalable spokes pTx pulses. Four volunteers (two males and two females) gave written consent in accordance with ethical approval from the Cambridge Human Biology Research Ethics Committee (HBREC.2020.270).

Manual B_0_ shimming was performed so the whole brain water linewidth was less than 50 Hz. Scout images were acquired in sagittal orientation, and TSE coronal slices were positioned perpendicular to the hippocampus. Per‐channel B_1_^+^ and ΔB_0_ maps were acquired with the vendor's adjustment sequences, which were then masked and resliced in the TSE slice direction with the scanner's built‐in PPD framework. The field maps were exported as a MatLab (MathWorks) .mat file, which was transferred to a workstation for pulse optimization (as described in the Pulse Design section 3.1 above).

CP mode TSE scans were acquired for reference with the following parameters: 75 ms TE, 8000 ms TR, 2.0 mm slice thickness, 0.5 mm in‐plane resolution, nine echo train length, and 15.0 ms echo spacing. Nominal refocusing flip angles were 120°, except for the first refocusing pulse, which was 150°. These parameters match the epilepsy presurgical protocol used at our site.^8^ The sequence timing allowed 54 slices per 8000 ms TR. However, due to the difference in power between CP and pTx mode allowed by the vendor (1 W per channel 8 W total power in pTx, 20 W total power in CP; more details in Discussion section 5), we chose to run the CP mode scan also at the 8 W limit. This resulted in 43 slices per 8000 ms TR restricted by SAR.

For pTx pulse optimizations, a specific energy‐dose (SED) constraint was computed as follows: The limiting per‐pulse SED was computed based on the TR and number of slices for the CP protocol (because this ran at just under 100% SAR for pTx sequences). This was divided by 0.74 (increased 1.35×), which was the energy reduction from the constant‐time minimum‐energy VERSE conversion for the 1.6 ms Gaussian subpulse. This SED target was set in the pulse designer, and the designs were repeated at different Tikhonov regularization values, gradually increasing the SED target until it was possible to reach a flip‐angle normalized RMS error (RMSE) of <10%. This was a choice based on the tradeoff between flip angle homogeneity and the strict power constraints (see Discussion section), and by drawing on previous results (see Yetisir et al.^22^) (Figure S3). The maximum possible number of slices for pTx acquisition was then calculated to be 21 (49% of CP protocol) for the same fixed 8000 ms TR. This fixed the pTx protocol and the pulse design constraints. All other sequence parameters were kept the same as the CP protocol.

For comparison, DSC scans were performed with the DiSCoVER work‐in‐progress package (Siemens Healthcare) using the same acquisition parameters as the scalable spokes pTx scans. The pulse optimization used the recommended parameters with a global RF shim initialization and two‐tissue optimization, which maximizes the echo intensities from gray matter and CSF.

For each of the three methods, one set of pulses was used for the whole imaging slab.

## 4 RESULTS

**FIGURE 2:**
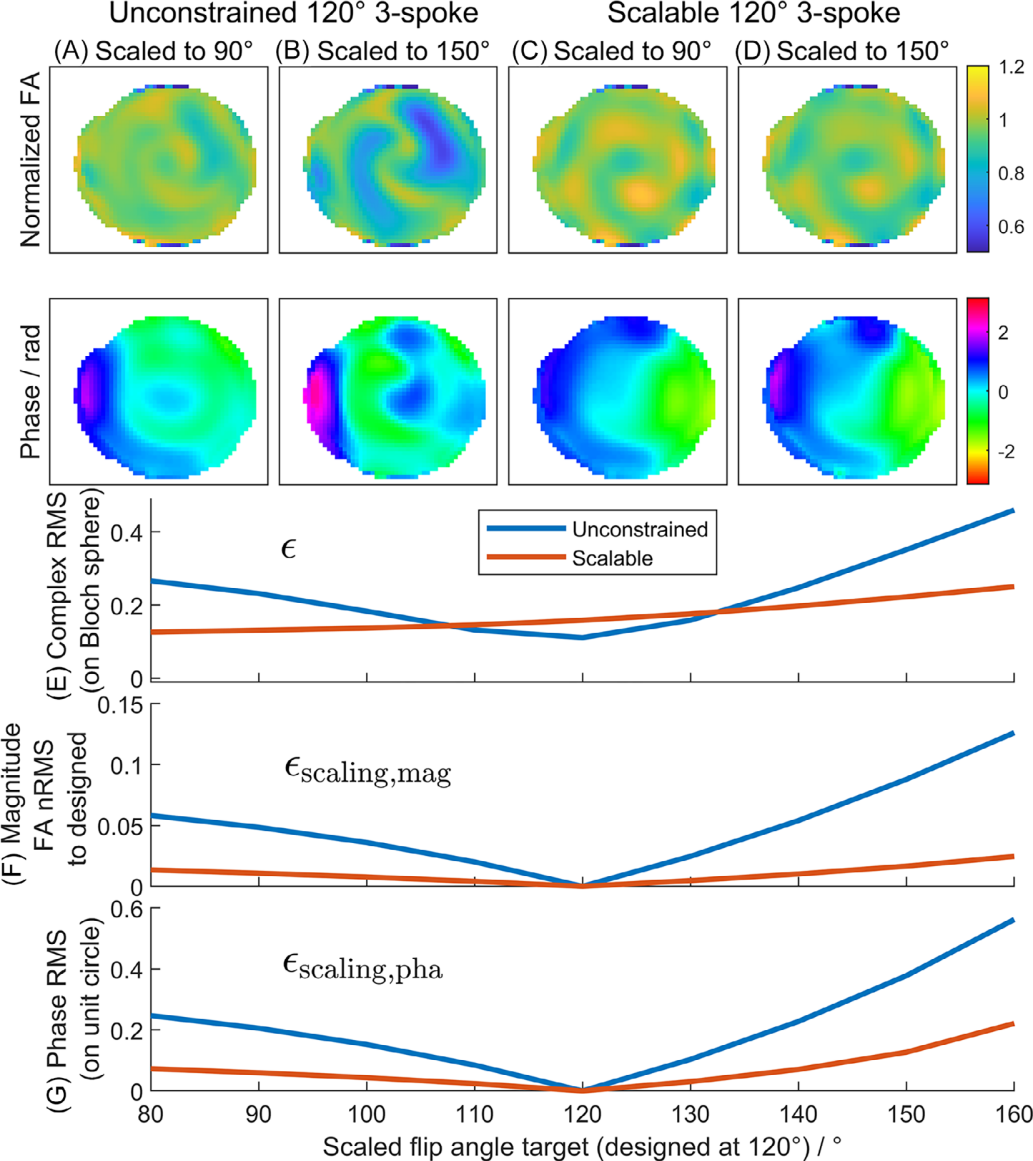
Scalability illustration with Bloch simulations. (A and B) The behavior of an unconstrained three‐spoke pulse designed without restrictions on the G_xy_ waveform symmetry. Scaling the voltage significantly changes the flip angle magnitude and phase patterns, whereas (C and D) show a three‐spoke pulse in which G_xy_ waveform symmetry was constrained. (E–G) Error component comparison between unconstrained and scalable three‐spoke pulses under scaling. Scalable three‐spoke pulses show smaller flip‐angle scaling errors in both magnitude and phase patterns.

The scaling performance of the scalable spokes pTx pulses is demonstrated in Figure 2 for 90° and 150° nominal flip angles but designed at 120°. The quantitative error metrics defined in Equations ((8), (9), (10))–((8), (9), (10)) are plotted for nominal flip angles from 80° (0.67×) to 160° (1.33×). As predicted by theory, the flip angle produced by a scalable three‐spoke pulse is closer to being proportional to the pulse voltage than for an unconstrained three‐spoke pulse. Figure 2A–D shows that the spatial profile of flip angle magnitude is consistent across voltages for the scalable pulse, whereas scaling the unconstrained pulse could produce dropouts. Crucially, the scalable pulse also maintained a more consistent spatial profile for phase, which means that in TSE imaging, the 90° phase difference required between excitation and refocusing pulses by the CPMG condition can be satisfied (mostly) across the whole pulse design volume by a single phase shift of the entire pulse. Figure 2E–G shows that upon scaling, the increase in flip angle error of the scalable spokes pulses was small compared to an unconstrained pulse, especially when a pulse is scaled down to lower target FA (lower peak voltage). The magnitude change was 1.3% (vs. 5.8% for the unconstrained pulse) across the phantom when scaled down to 0.67× and 2.5% (vs. 12.6% for the unconstrained pulse) when scaled up to 1.33×. When scaled down to 0.67× the phase error was 7.3% (vs. 24.7% for the unconstrained three‐spoke pulse) for the scalable pulse and 22.1% (vs. 56.2% for the unconstrained three‐spoke pulse) when scaled up to 1.33×.

The rotation axes were generally close to transversal, with an RMS deviation of 9.2° across all voxels for our scalable three‐spoke designed for 120° (Figure S2). The movement of the rotation axis upon scaling was different between voxels.

**FIGURE 3:**
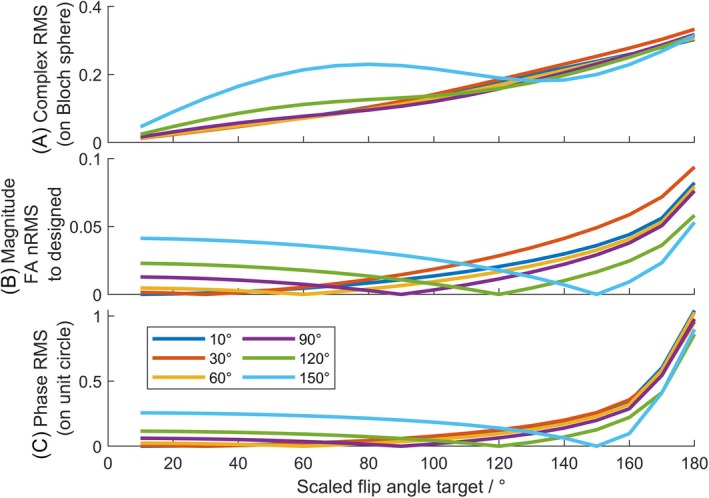
Pulse error quantities (as in Figure 2) plotted for scalable pulses each designed for a different flip angle (shown in the legend). (A) Magnetization error versus using target flip angle (Equation (10)). (B) Flip‐angle magnitude versus the pattern at the design flip angle (Equation (8)). (C) Phase errors (Equation (9)).

Figure 3 shows a similar plot but for different flip angles at which the scalable pulse was designed. As expected, the scaling errors increase as the flip angle steps away from the designed value, and the error generally increases faster when scaling up. Therefore, the best target design angle would be close to the center of the range of flip angle to use in the sequence. However, it should be noted that high design flip angles (≥150°) introduced a significantly higher error when scaling down, likely limited by the quality of our specific STA pulse optimizer close to 180°.

**FIGURE 4:**
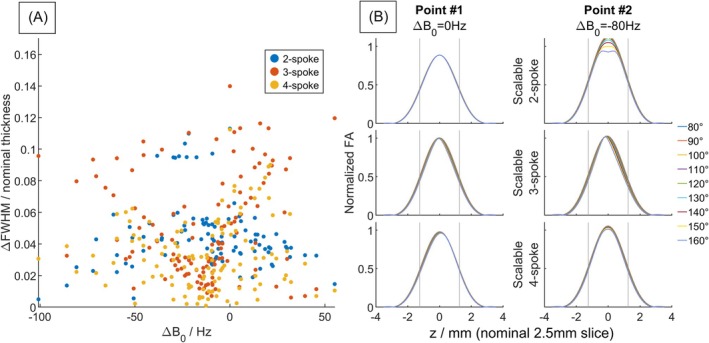
Slice profile comparison between scalable two‐, three‐ and four‐spoke pulses from Bloch simulations using in vivo field maps. (A) Scatter plot from 122 voxels (3×3 downsampled from within the brain to reduce clutter) showing the relationship between ΔB_0_ offset and the corresponding maximum deviation in FWHM across a ±33% flip‐angle scaling range at each voxel. (B) Example slice profiles for each pulse type from two voxels having different ΔB_0_ offsets. Each plotted line represents a different scaled target flip angle ranging from 80° to 160°. The design target (i.e., unscaled pulse) was 120°. The nominal slice thickness (2.5 mm) is labeled with gray lines.

Figure 4 shows simulated slice‐profile comparison of scalable two‐, three‐ and four‐spoke pulses at two points with different ΔB_0_ in brain. All pulses showed certain variations in the slice profile when scaled, the degree of which show no obvious pattern in relation to ΔB_0_. The scalable three‐spoke pulses showed slightly higher spread in the achieved slice thickness, and more asymmetry in the slice profile distortion, which perhaps reflects that we chose not to use a time‐antisymmetric slice‐selection gradient waveform. Across a grid of 100 voxels, the FWHM percentage spread (max–min thickness across the scaled flip angle range, normalized by the nominal slice thickness) averages to 4.6%, 5.1%, and 2.9% (median 4.2%, 4.1%, and 3.0%) for two‐, three‐ and four‐spoke pulses, respectively.

**FIGURE 5:**
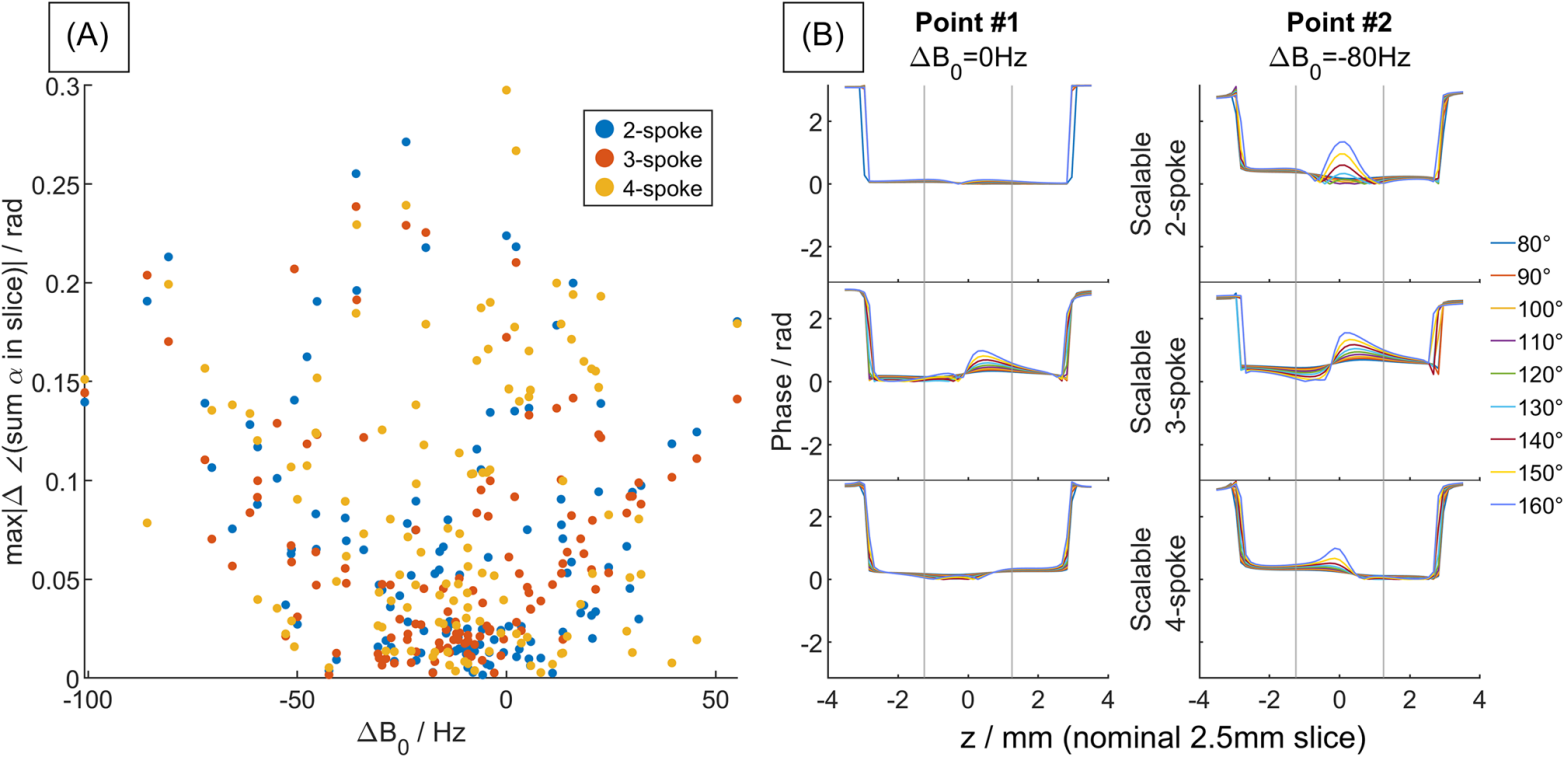
Similar plot as Figure 4 but showing the phase across the slice direction. (A) Scatter plot of the maximum change in phase (of the complex flip angle summed through slice) when scaled between 80° and 160° for each voxel, comparing between two‐, three‐, and four‐spoke scalable pulses. (B) The variation of phase through slice for two voxels with different ΔB_0_ offsets. Nominal thickness (2.5 mm) is labeled with gray lines. This variation through slice comes mostly from the first‐order terms in average Hamiltonian theory. When ΔB_0_ is small, the effect can cancel out with even symmetric spokes.

The phase variation through slice compared between scalable two‐, three‐, and four‐spoke pulses is shown in Figure 5. The maximum phase variation of the complex flip angle (summed through slice) across the scaled flip angle range, averaged across the slice, is 0.066, 0.063, and 0.084 radians (median 0.045, 0.047, 0.064 radians) for two‐, three‐, and four‐spoke pulses, respectively.

**FIGURE 6:**
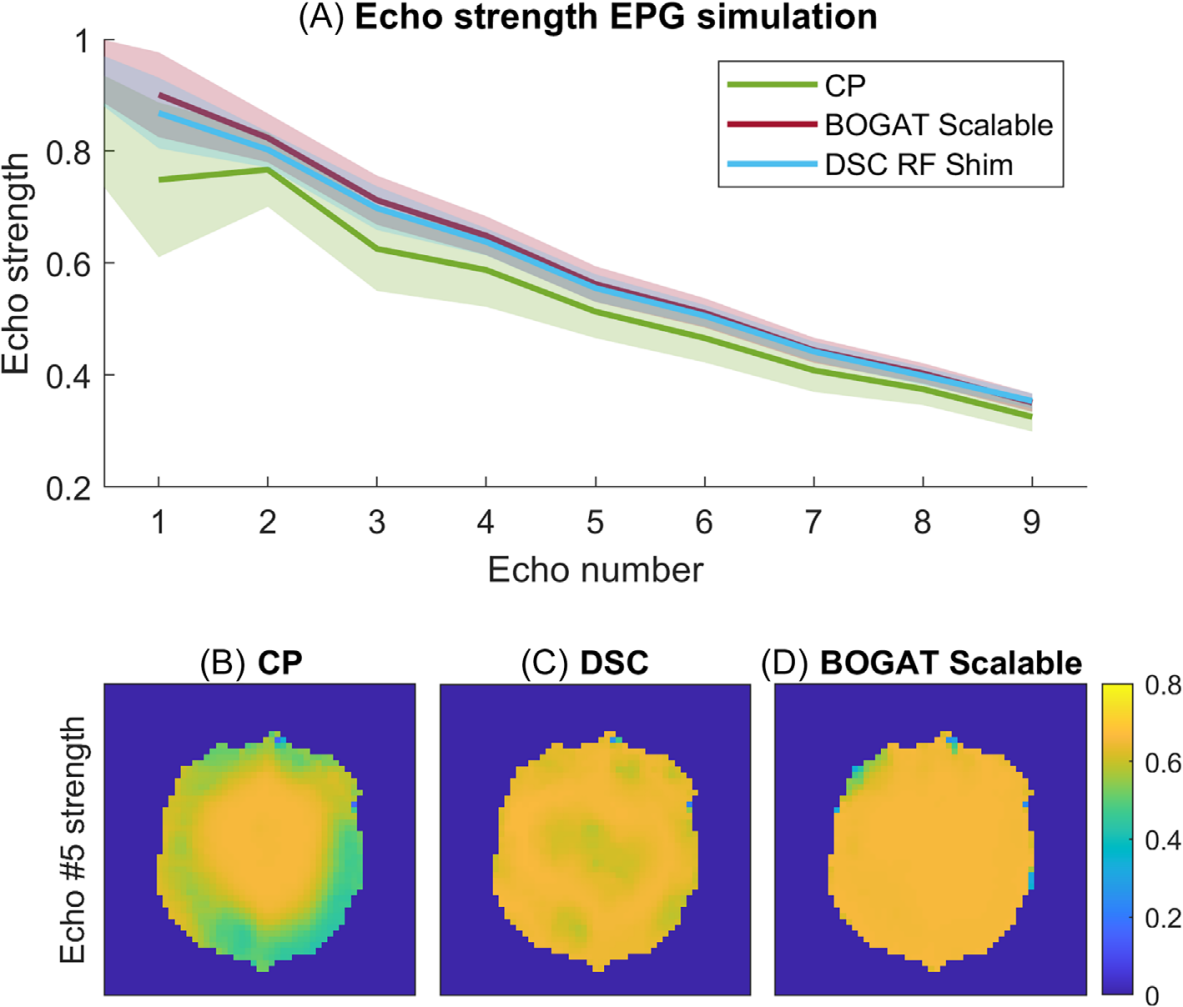
TSE strengths simulated by EPG, comparing the performance between CP, direct signal control RF shimming optimization, and our BOGAT scalable three‐spoke pulses. (A) Echo amplitudes plotted as mean and SD across all voxels in this slice. (B–D) Amplitude of the central (fifth) echo for each method. BOGAT, Bayesian optimization of gradient trajectory; CP, circularly polarized; EPG, extended phase graph.

Figure 6A presents EPG simulations of the TSE echo train using in vivo field maps. The scalable three‐spoke pulse performed comparably with DSC shimming optimization for the mean echo amplitudes at the center echo. Figure 6B–D shows the spatial profiles for the central (fifth) echo in a typical slice with relatively strong ΔB_0_ effects due to the proximity to frontal sinus. Scalable three‐spoke pulses and DSC RF shimming both performed significantly better than CP mode (echo transverse magnetization 0.563 ± 0.030, 0.556 ± 0.025, 0.514 ± 0.047, respectively).

**FIGURE 7:**
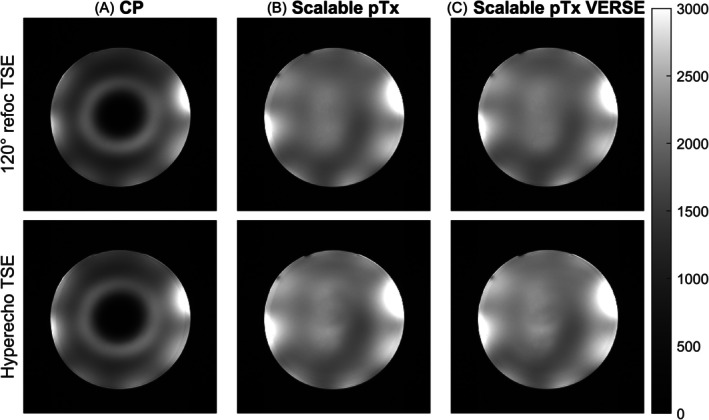
(A) TSE acquisitions on the UK7T study's uniform spherical agar phantom with (A) CP, (B) scalable three‐spoke pTx pulses designed with BOGAT, and (C) the scalable pulse with additional fixed‐time variable‐rate selective excitation (VERSE). Two types of echo trains were tested: A 120° constant refocusing train (top), and a variable flip‐angle hyperecho refocusing train (bottom).

Figure 7 shows TSE images acquired with the scalable pulses in the UK7T study's agar phantom.^45^ The three‐spoke pulse images show better uniformity than CP images, both for the constant refocusing train and for the variable flip‐angle hyperecho refocusing train. Applying VERSE reduced SAR by 26% without an observable impact on image quality. The pulse designs without VOP constraints took on average 9 s per slab.

**FIGURE 8:**
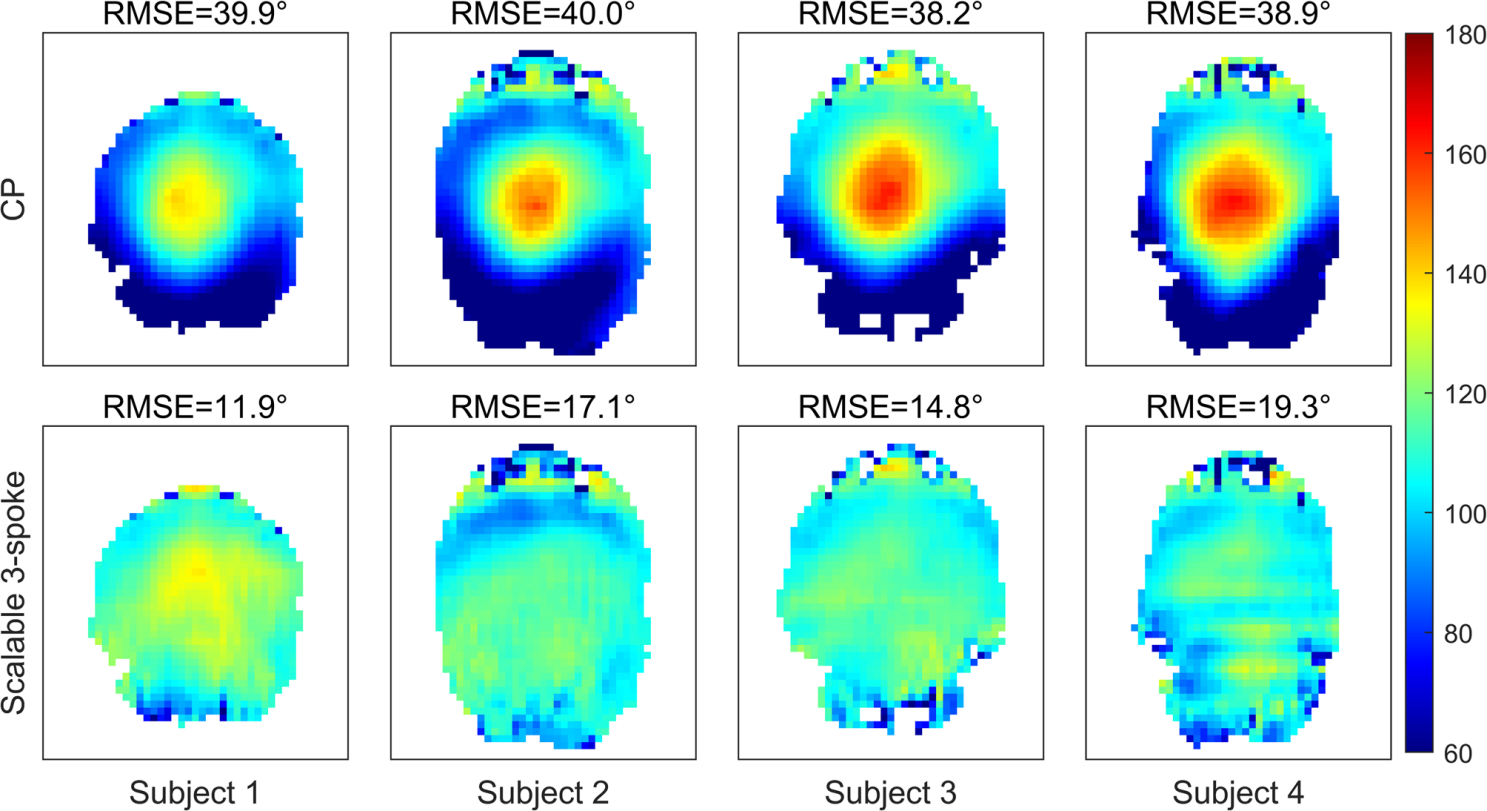
Simulated flip angle comparisons between (A) the CP pulse and (B) the BOGAT‐designed scalable three‐spoke pTx pulse (before VERSE) in all four subjects. Slices were planned perpendicular to the hippocampus (approximately coronal). Masks are generated from the MP2RAGE images. Pulse designs used 120° target flip angle. The three‐spoke pulses show better flip angle homogeneity. Some artifacts for subject 4 was due to the (vendor‐provided) field maps that were acquired in the transverse direction and interpolated to coronal slices.

For hippocampus imaging, Figure 8 shows the simulated flip angle patterns and errors of the pTx scalable three‐spoke pulses compared to the default CP pulses. The scalable spokes pTx pulses achieved significantly better homogeneity than the CP pulse, albeit over a smaller number of slices due to the difference in pulse energy under SAR constraints on Terra scanners (Siemens Healthineers) (see the Methods section). A summary of the flip angle errors for the designed 120° pulse as well as the scaled 90° and 150° pulses were compared to CP and are shown in Table 1. The scalable spokes pTx pulses improved the normalized flip angle RMSE by 52% (23.0% to 10.1%), 56% (22.3% to 10.6%), and 50% (23.0% to 11.4%), respectively ($\left({\text{RMSE}}_{\mathrm{CP}}-{\text{RMSE}}_{\mathrm{pTx}}\right)/{\text{RMSE}}_{\mathrm{CP}}$), and the errors were consistent when scaled. The pulses for all subjects were limited by the 1 W per channel, 8 W total power limit (enforced as a VOP SED constraint), which represents a 104% increase of SED per slice from the CP protocol. The total power deposition per slice increased by 84% (SD 14%). In vivo pulse designs with VOP constraints required 45 s per slab on average.

**TABLE 1:** Flip angle normalized RMSE in all subjects compared between CP and scalable three‐spoke pTx pulses before and after variable‐rate selective excitation (VERSE).

	Target/°	Flip angle normalized RMSE/%				
		Subject 1	Subject 2	Subject 3	Subject 4	Mean
CP	90	22.3	26.2	21.6	21.8	23.0
	120	21.9	26.0	20.7	20.5	22.3
	150	22.3	26.3	21.0	22.5	23.0
Scalable three‐spoke	90 (scaled)	7.8	10.7	9.9	12.1	10.1
	120 (designed)	7.9	11.3	10.3	12.8	10.6
	150 (scaled)	8.2	12.4	11.0	14.0	11.4
Scalable three‐spoke VERSE	90 (scaled)	8.5	11.4	12.9	13.2	11.5
	120 (designed)	8.6	11.9	13.3	13.5	11.8
	150 (scaled)	8.8	12.5	14.0	13.9	12.3

*Note*: The CP pulses reference voltage accounts for the per‐subject reference voltage. The pTx pulses were designed for 120° nominal flip angle and scaled to 90° for excitation and 150° for the first refocusing, and were kept at 120° for subsequent refocusing pulses.

Abbreviations: CP, circularly polarized; pTx, parallel transmit; RMSE, RMS error.

**FIGURE 9:**
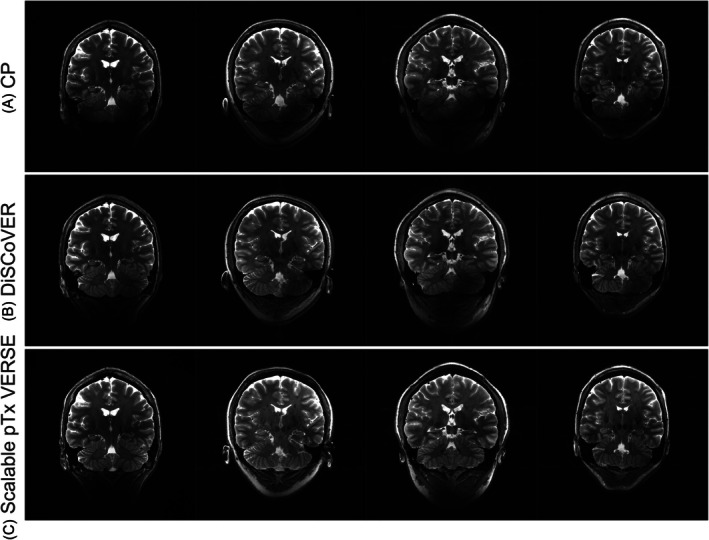
In vivo TSE images acquired perpendicular to hippocampus with (A) CP, (B) DiSCoVER and (C) scalable 3‐spoke pTx pulse with VERSE. Ringing artifacts in (B) column 3 is due to subject motion.

Figure 9 compares the T_2_‐weighted TSE images acquired in all subjects between CP, DiSCoVER (Siemens Healthcare), and pTx scalable spokes acquisitions. The CP images show complete dropouts in parts of the cerebellum. This extends into the left inferior temporal lobe, causing some contrast loss. DiSCoVER images recover most of the signal, with the exception of some left–right asymmetry in the cerebellum. Scalable spokes pTx images show better fidelity throughout the brain. This observation is consistent for all four subjects.

## 5 DISCUSSION

We have introduced scalable spokes pTx pulses for slice‐by‐slice 2D imaging. They achieve good scaling properties demonstrated through simulations and TSE imaging experiments. The scaling errors show comparable behavior to those of the 3D k_T_‐point pulses shown previously,^24^ specifically that the error curves increase more rapidly when scaling the flip angles up for a large amount. However, because the scaling quality decreases with longer pulse duration, the 2D pulses show slightly higher scaling errors than their 3D counterparts, due to need for longer subpulses (1‐2 ms for slice‐selective pulses compared to ˜0.2 ms for hard pulses). When the refocusing target is close to 180°, high flip angle–based design approaches (e.g., method in Yetisir et al.^22^) may achieve better flip angle error.

Our scalable three‐spoke pTx pulse implementation validates our average Hamiltonian theory result that slice‐selection gradients G*_z_* can be time‐symmetric, whereas in‐plane gradients G_*xy*_ are time‐antisymmetric. This allows a practical total pulse duration. Simulations showed a small increase in the slice‐profile error under scaling (5.1% spread in FWHM), which corresponds to (at most) a 0.13 mm difference for a 2.5 mm slice thickness for an average voxel across a ±33% flip‐angle scaling range, which we consider an acceptable tradeoff.

Enforcing a time‐symmetric RF waveform increased the relative weights applied to the non‐DC spoke (i.e., first and second spokes in the three‐spoke pulse) compared to the center DC spoke (i.e., second spoke in the three‐spoke pulse). This is because the same RF shim is applied twice with the same polarity in the pulse, whereas the magnitude least squares Tikhonov term has the same regularization effect on the amplitude of each one subpulse. This makes it particularly important to compensate for RF‐gradient timing delays, which affects all bipolar spokes pulses, to minimize phase errors. From our testing and simulations, a phase shift of as little as 10° could completely distort the resulting flip angle pattern. Therefore, it is imperative to correct for the timing delay and the eddy current effects of the spokes slice‐selection gradients prior to using these pulses as explained in the Methods section.

Whereas hippocampus TSE imaging using the scalable spokes pTx pulse produced higher uniformity and quality, the protocol is limited by SAR. The vendor's current limit for the Nova 8Tx32Rx head coil on 7 T Terra MRIs (Siemens Healthineers) is 1 W per channel, 8 W total power in pTx in the IEC first level mode, whereas in CP mode that is restricted to 20 W total power. We have previously tested sequences using (nonscalable) spokes pTx pulses by visiting other sites to access an 8Tx head coil with full VOP SAR supervision for a study on diffusion imaging.^17^ Those results suggested that the pulse design parameters at the limit of the conservative per channel SAR constraints of the Nova head coil could leave as much as 50% to 60% SAR headroom for a similar coil with full VOP model for SAR supervision as opposed to simple power limits.

Recognizing these limits and the likelihood for them to be alleviated in future, we prioritized demonstrating the potential that scalable spokes pTx pulses have in terms of higher fidelity imaging. Our choice of homogeneity target (<10% flip angle error at 120°) increased the average pulse energy compared to CP sequence. On our current 7 T scanner, we recognize a limitation that the number of slices covered by our pTx scans was approximately 50% of that possible using CP pulses. In future studies, it would be interesting to explore the performance SAR tradeoff on coils with full electromagnetic‐modeled VOP supervision.

Whereas we chose to demonstrate the application of the scalable spokes pTx pulses for TSE imaging, the use case could be extended. For instance, they could be applied to several 2D sequences requiring multiple different flip angles in an imaging protocol, recognizing that the slice‐by‐slice designs of 2D pulses could be time‐consuming.

We chose to compare our scalable pulses in TSE imaging with DSC optimizations because we wanted to evaluate its performance against a popular, more mature solution. It should, however, be noted that these methods do not exploit the same degrees of freedom in the pTx system. The spokes pulses use more DOFs in a single pulse to achieve a better flip angle pattern, whereas DSC uses DOFs along the echo train to improve the average echo homogeneity. The three‐spoke pulses can achieve more uniform images with the tested parameters, whereas DSC has a theoretical advantage to achieve higher, more uniform signal over a longer echo train due to the increased DOFs. In future research, it could be beneficial to combine these methods, applying scalable pulses as part of a DSC‐optimized flip angle train, which might achieve even better TSE image quality or allow more judicious use of the SAR budget.^49^

## 6 CONCLUSIONS

Scalable spokes pTx pulses produce flip angles that vary approximately linearly with peak voltage while maintaining consistent spatial patterns of phase. They achieve better flip‐angle homogeneity than CP pulses or RF shimming. Scalable spokes pTx pulses are therefore well suited for 2D TSE imaging at 7 T, especially because the phase consistency enables compliance with CPMG conditions across the volume of interest. We demonstrate this in an agar phantom and in four volunteers. With a choice of <10% flip angle RMSE for scalable spokes pulse designs, current vendor‐provided SAR limits on our 7 T Terra MRI (Siemens Healthineers) restrict coverage to ˜50% of the slices accessible by a CP scan of equal duration.

## FUNDING INFORMATION

M.Z. is supported by the Medical Research Council (MR N013433‐1) and the Cambridge Trust. C.T.R. acknowledges research support from Siemens. This research was supported by the National Institute for Health and Care Research (NIHR) Cambridge Biomedical Research Centre (BRC‐1215‐20014). The Cambridge 7 T MRI facility was cofunded by the University of Cambridge and the Medical Research Council (MR/M008983/1).

## CONFLICT OF INTEREST STATEMENT

C.T.R. acknowledges research support from Siemens.

## Supporting information


**Figure S1.** The effect of (A) a single spoke subpulse and associated slice select gradient can be pictured as (B) an instantaneous effective rotation in the interaction picture, according to Average Hamiltonian Theory. (C) When constructed as a self‐refocused pulse, it is produces an effect in the initial rotating frame (D) of the instantaneous rotation sandwiched between B0 free precessions of half of the subpulse duration, proven in Gras et al.^24^ This concept can be applied to (E) the full 3‐spoke pulse where prephasing and rephasing gradients cancel out within the pulse, ultimately yielding (F) a series of RF and gradient events equivalent to a 3D non‐selective pulse scenario.
**Figure S2.** (A) Illustration of the change in rotation axis when the scalable 3‐spoke pulse designed for 120° was scaled between 80° and 160°. Each trace shows how the rotation axis change for one voxel (six shown). (B) RMS deviation from a pure transverse rotation axis over all voxels in the phantom. (C) RMS deviation from the designed rotation axis over all voxels in the phantom.
