# Supplementary material for: Scalable spokes pTx pulses for 2D turbo‐spin‐echo imaging at 7 T
Source: Magn Reson Med. 2025 Sep 29;95(2):740–55. doi: 10.1002/mrm.70068 (PMC12681301; doi:10.1002/mrm.70068)
Supplement: Supplementary file 1 — Figure S1. The effect of (A) a single spoke subpulse and associated slice select gradient can be pictured as (B) an instantaneous effective rotation in the interaction picture, according to Average Hamiltonian Theory. (C) When constructed as a self‐refocused pulse, it is produces an effect in the initial rotating frame (D) of the instantaneous rotation sandwiched between B0 free precessions of half of the subpulse duration, proven in Gras et al. 24 This concept can be applied to (E) the full 3‐spoke pulse where prephasing and rephasing gradients cancel out within the pulse, ultimately yielding (F) a series of RF and gradient events equivalent to a 3D non‐selective pulse scenario. Figure S2. (A) Illustration of the change in rotation axis when the scalable 3‐spoke pulse designed for 120° was scaled between 80° and 160°. Each trace shows how the rotation axis change for one voxel (six shown). (B) RMS deviation from a pure transverse rotation axis over all voxels in the phantom. (C) RMS deviation from the designed rotation axis over all voxels in the phantom. [file MRM-95-740-s001.docx]

# Supplementary information


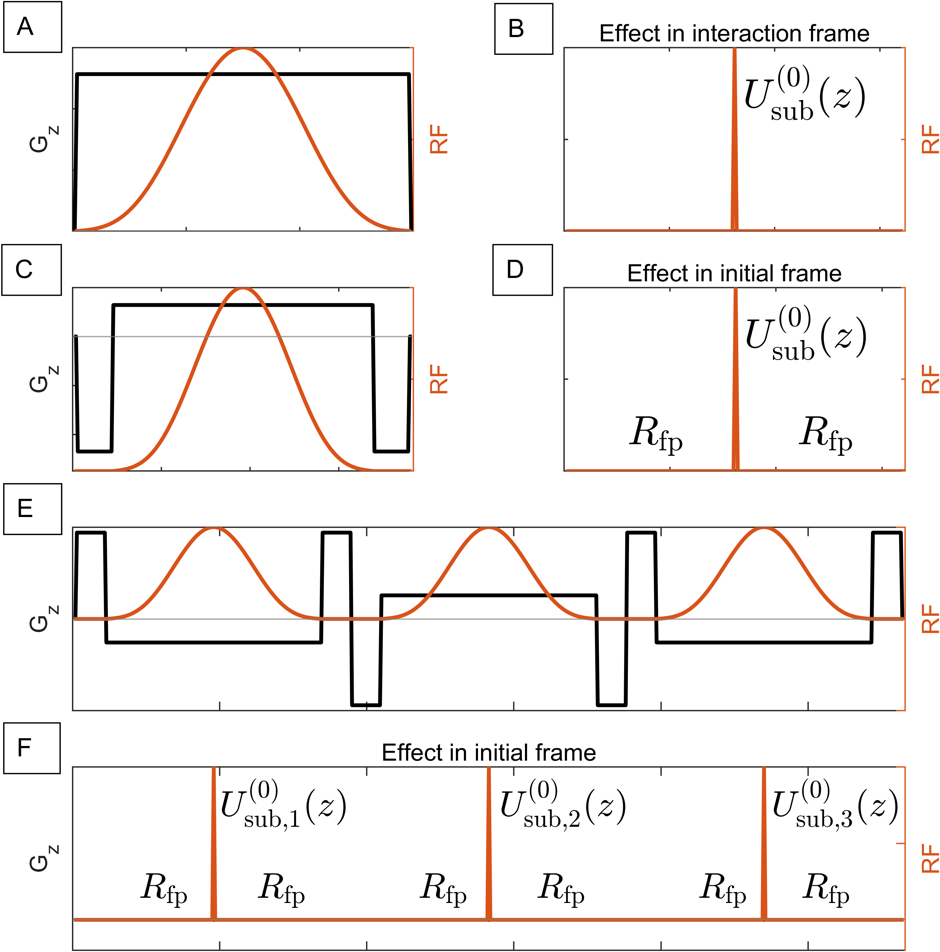


Figure S1 The effect of (A) a single spoke subpulse and associated slice select gradient can be pictured as (B) an instantaneous effective rotation in the interaction picture, according to Average Hamiltonian Theory. (C) When constructed as a self-refocused pulse, it is produces an effect in the initial rotating frame (D) of the instantaneous rotation sandwiched between B0 free precessions of half of the subpulse duration, proven in Gras et al.^24^ This concept can be applied to (E) the full 3-spoke pulse where prephasing and rephasing gradients cancel out within the pulse, ultimately yielding (F) a series of RF and gradient events equivalent to a 3D non-selective pulse scenario.


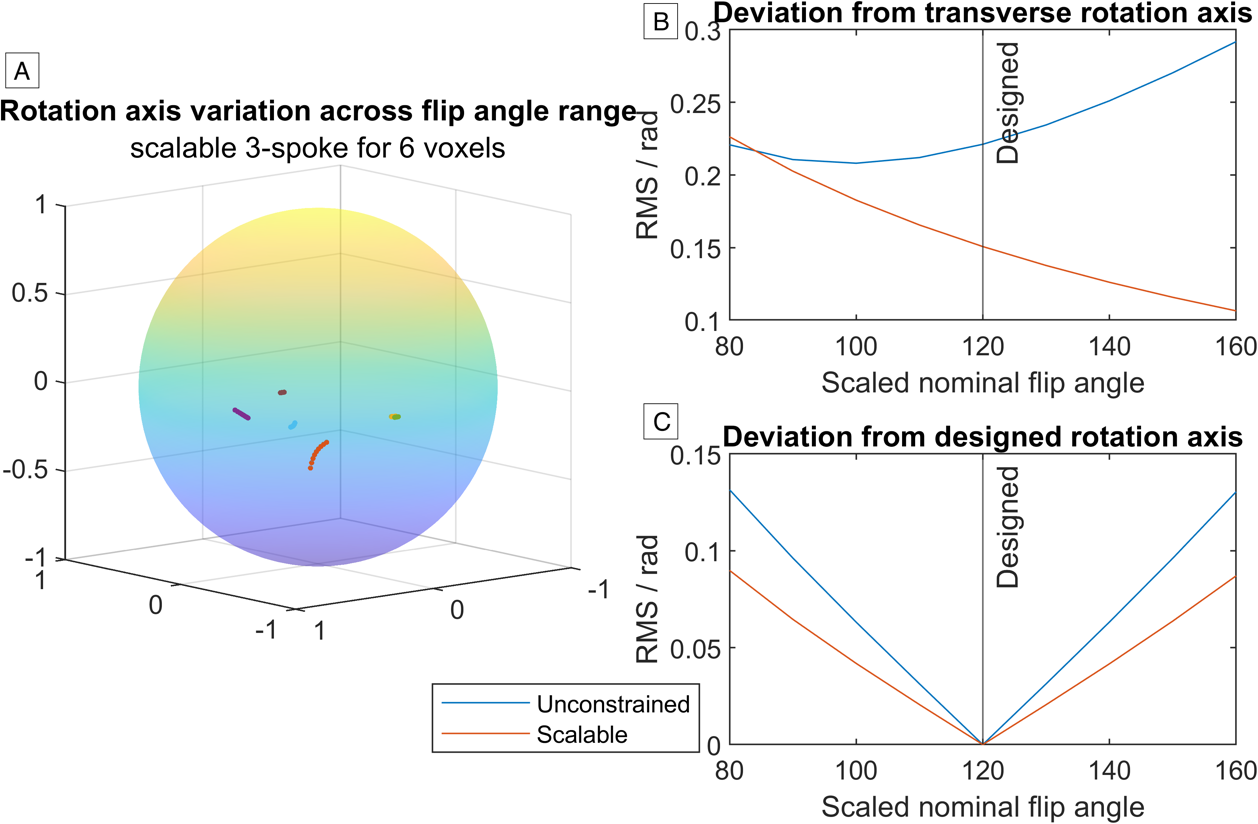


Figure S2 (A) Illustration of the change in rotation axis when the scalable 3-spoke pulse designed for 120° was scaled between 80° and 160°. Each trace shows how the rotation axis change for one voxel (six shown). (B) RMS deviation from a pure transverse rotation axis over all voxels in the phantom. (C) RMS deviation from the designed rotation axis over all voxels in the phantom.
